# “It's like I used to share a room with self‐injury, but now it lives next door”: Exploring experiences of naturalistic improvement in non‐suicidal self‐injury

**DOI:** 10.1111/papt.12567

**Published:** 2024-12-19

**Authors:** E. Hudson, S. Hartley, P. J. Taylor

**Affiliations:** ^1^ Division of Psychology and Mental Health, School of Health Sciences University of Manchester, Manchester Academic Health Sciences Centre Manchester UK; ^2^ Greater Manchester Mental Health NHS Foundation Trust Manchester UK; ^3^ Child and Adolescent Mental Health Service Pennine Care NHS Foundation Trust Ashton‐Under‐Lyne UK

**Keywords:** NSSI, qualitative, recovery, self‐injury

## Abstract

**Background:**

Many people who engage in non‐suicidal self‐injury (NSSI) do not access support from health services, and evidence regarding the effectiveness of interventions is mixed. Despite this, NSSI prevalence rates decrease from adolescence into adulthood. Little is known about what helps alleviate difficulties with NSSI beyond psychological or medical intervention. This study sought to understand factors influencing naturalistic improvements in NSSI.

**Methods:**

Semi‐structured interviews were conducted over video call with 16 participants who believed their difficulties with NSSI had improved due to factors not attributed to psychological or medical intervention. Interviews were audio recorded and transcribed verbatim for analysis.

**Results:**

A reflexive thematic analysis revealed four main themes. Increased insight into NSSI experiences promoted self‐compassion and self‐acceptance and enabled participants to reflect on the conflicting role of NSSI. Safe and supportive relationships helped alleviate loneliness, and developing alternative coping strategies enhanced feelings of control over self‐injury. Creating a life guided by personal values promoted independence, choice, and self‐esteem.

**Conclusions:**

The findings of the study highlight several internal and external naturalistic processes deemed meaningful in improving difficulties with NSSI. Clinical implications include the importance of developing and embedding these approaches within services and interventions to improve outcomes for individuals who self‐injure while promoting a person‐centred approach.

## INTRODUCTION

Non‐suicidal self‐injury (NSSI) is the act of inflicting intentional damage to one's own body without suicidal intent (Klonsky, 2007). Estimates of lifetime prevalence rates of NSSI range from 4.9% to 17% among adults (Klonsky, 2011; Liu, 2023; Swannell et al., 2014; Whitlock et al., 2006). Engaging in NSSI can have a wide range of intrapersonal and interpersonal functions (Reinhardt et al., 2021), and research suggests NSSI is most frequently used to regulate distressing emotions or internal states (Klonsky, 2007; Klonsky et al., 2014; Lewis & Heath, 2015; Taylor et al., 2018). NSSI has several physical consequences, such as the risk of infection and scarring (Cassels & Wilkinson, 2016), and is a risk factor for later suicidal behaviour (Chartrand et al., 2022; Ribeiro et al., 2016). Additionally, NSSI is associated with a variety of mental health difficulties and psychological distress (Bentley et al., 2014; Daukantaitė et al., 2021; Klonsky et al., 2003) and can have detrimental impacts on family and peer relationships (De Luca et al., 2022; Tan et al., 2014). Less is known about how people recover from or overcome NSSI (Andover, 2012), indicating a need to further understand factors that improve difficulties with NSSI to inform meaningful interventions.

Within the literature and clinical interventions, NSSI recovery is frequently conceptualised as the reduction or cessation of self‐injuring behaviours (Calati & Courtet, 2016; Grunberg & Lewis, 2015; Kress & Hoffman, 2008; Turner et al., 2014). The Trans‐Theoretical Model of Change (Prochaska & DiClemente, 1982) has been applied to NSSI, outlining how recovery may progress through a series of stages from pre‐contemplative (i.e., no need for change is recognised) through to action (i.e., taking steps to change) and maintenance/relapse prevention (Kamen, 2009; Kruzan & Whitlock, 2019). Whilst a useful framework, the focus here is primarily on reduction and cessation of the behaviour. More recently, person‐centred approaches have acknowledged that recovery extends beyond cessation and is an ongoing, multifaceted process (Bradley et al., 2023; Lewis et al., 2019; Lewis & Hasking, 2020). Research has alluded to naturalistic recovery processes in broader explorations of mental health recovery, whereby factors influencing recovery extend beyond psychological or medical interventions. Onken et al. (2002) found recovery among 115 participants with a range of psychiatric diagnoses was attributed to dynamic interactions between various environmental and inter‐ and intrapersonal domains. This included access to basic resources (e.g., liveable income, affordable housing), engaging in meaningful activity, developing self‐acceptance and a sense of purpose, building positive social relationships, and having increased independence and choice. As the term ‘recovery’ may place NSSI within a disease‐based framework and increase stigma (Hasking & Boyes, 2018; Lewis & Hasking, 2020), the current study will use the term ‘improvement’ to indicate perceived holistic improvements with NSSI‐associated difficulties.

Research shows NSSI typically peaks around adolescence but decreases in early adulthood (Plener et al., 2015), suggesting many young people who engage in self‐injury subsequently may overcome it (Cassels & Wilkinson, 2016). There is evidence some interventions, including Dialectical Behaviour Therapy (DBT), may be helpful in reducing NSSI (Cook & Gorraiz, 2016; Turner et al., 2014). However, a meta‐analysis by Calati and Courtet (2016) found no evidence of efficacy for a range of psychotherapies in reducing NSSI. Nonetheless, many individuals who engage in NSSI do not access formal psychological therapy or support from healthcare services (Deliberto & Nock, 2008). This could be due to barriers including stigma, shame, pejorative, or unhelpful responses from others following disclosure (e.g., hostility, silence, avoidance), or perceived iatrogenic harm from engaging with services (Rosenrot & Lewis, 2020; Sykes et al., 2015). Many individuals who self‐injure may therefore follow a more naturalistic course of recovery, whereby factors beyond psychological or medical intervention may improve difficulties with NSSI.

Research has started to explore naturalistic recovery processes in NSSI. For example, in a sample of participants who reduced or ceased NSSI without psychological or medical intervention, Buser et al. (2014) found participants reduced NSSI following recognition of the serious physical harm from self‐injury, meeting disapproval or acceptance from others, and moving from unhealthy to healthy surroundings. Similarly, Shaw (2006) found participants attributed NSSI cessation to educational and career‐related achievements and support from social networks, in addition to valuing professional support. However, these studies focused on contributors to NSSI cessation (Buser et al., 2014; Shaw, 2006), as opposed to adopting a broader understanding of improvement in NSSI that is not limited to behavioural cessation (Lewis et al., 2019; Lewis & Hasking, 2020).

There is a paucity of research into the idea of personal naturalistic improvements in NSSI, focusing on people who have experienced improvements in their NSSI difficulties that are not attributed to psychological or medical intervention. Improved understanding of this phenomenon may highlight important mechanisms for change that could subsequently inform health services. The current study aims to investigate individual experiences of naturalistic improvements in NSSI. Improvements with NSSI will be defined broadly to allow greater diversity of participant experiences. Qualitative research was appropriate as the study aims to enhance understanding of personal experiences and processes (Harper & Thompson, 2012).

## METHOD

### Design

Ethical approval was granted from a University Research Ethics Committee. A cross‐sectional qualitative interview study design was used. This paper follows the Consolidated criteria for Reporting Qualitative Research (COREQ; Tong et al., 2007). A summary of the COREQ items and where these are met is included in Table S1.

### Participants

Recruitment took place between January 2022 and August 2022. Participants were recruited through third‐sector organisations, social media (Twitter) and universities in Northern England via adverts and email bulletins. Snowball sampling was conducted where participants were asked to share the recruitment poster with other individuals that may meet the inclusion criteria. Several recruitment avenues were used to increase the likelihood of accessing individuals with a range of experiences, at different stages of their ‘recovery’ journey. Inclusion criteria were: (1) individuals must be aged 16 years or over; (2) they must have engaged in intentional self‐inflicted bodily damage without suicidal intent, occurring on five or more days within a one‐year period (this could be any one‐year period in their lives); (3) the individual believed their difficulties with NSSI had improved in some way; (4) the individual did not attribute this improvement to psychological therapy or medication; and (5) the individual must understand verbal and written English. Criteria were assessed by self‐report during the initial screening appointment.

The NSSI diagnostic criteria adapted from the DSM‐5 (American Psychiatric Association, 2013) was used to ensure and operationalise that NSSI had been a significant difficulty, and the nature of the study was relevant to potential participants. As the study was interested in individuals who believed their difficulties with NSSI had improved, this was open for individuals to self‐define and decide if they met this criterion. Individuals were eligible if they responded affirmatively to the question, “Do you feel your difficulties with self‐injury have improved in some way?”. Individuals who had accessed therapy or medication were still eligible if they did not attribute improvements in their NSSI to these treatments. Individuals experiencing current suicidal ideation with a plan or active intent to end their life or a plan to engage in medically serious self‐injury (e.g., ligaturing, overdose) were excluded. This was determined by the researcher during the screening appointment.

### Measures

#### Demographic information

The demographic information sheet collected information regarding participants' age, ethnicity, employment, education, and health service access history.

#### Self‐injurious thoughts and behaviours interview (SITBI)

The NSSI subsection of the SITBI was used to identify participants' NSSI history. The SITBI is a structured interview that assesses the frequency and characteristics of self‐injurious thoughts and behaviours (Nock et al., 2007). The SITBI has good validity and reliability and is widely used in NSSI research (Nock et al., 2007).

#### Questionnaire about the process of recovery (QPR)

The QPR was used to collect information relating to participants' personal recovery. The QPR is a 15‐item self‐report measure of recovery developed collaboratively between clinicians and service user researchers (Neil et al., 2014). The QPR has adequate psychometric properties across populations with a range of mental health diagnoses (Williams et al., 2015). QPR scores range from 0 to 60, with higher scores indicating greater levels of personal recovery.

### Interviews

A topic guide containing open‐ended questions was developed with individuals with lived experience of mental health difficulties and NSSI to guide discussions in the semi‐structured interviews. This was done via patient and public involvement and consultation meetings with a pre‐existing group set up to advise on mental health‐related projects and a one‐to‐one meeting with a lived experience mental health consultant. This led to various changes, including the decision not to use the word “recovery” in the interviews given criticisms of how this word has sometimes been used within health services. Interviews were conducted by the lead researcher, and the topic guide covered areas exploring experiences of NSSI improvement (e.g., what helped you reach this point with your self‐injury?), experiences or perceptions of health services (e.g., what is your experience of support with self‐injury from health services?), and personal understandings of NSSI improvement or recovery (e.g., what does ‘recovery’ look like for you?). The topic guide was used flexibly to guide discussions, with questions and prompts adapted in response to previous interviews as the study progressed, whilst adhering to the same structure and topics. Interviews lasted from 23 to 72 min (*M =* 44 min). All participants chose to be interviewed via video call. For data analysis, interviews were audio recorded and transcribed verbatim.

### Procedure

Individuals that expressed interest in taking part were provided with the participant information sheet and invited to a screening appointment over video call or telephone to confirm eligibility. Following this, eligible individuals were invited to the interview appointment and were offered a choice of meeting face‐to‐face at the university or over video call on Zoom. After obtaining consent at the beginning of the interview appointment, participants completed the demographic information sheet and emergency contact sheet consistent with the risk protocol. Participants completed the SITBI (Nock et al., 2007) and the QPR (Neil et al., 2014) verbally with the researcher before proceeding to the interview. Following the interview, participants were verbally debriefed and provided an e‐voucher for their participation. Participants that consented during the initial consent process were invited to a further member‐checking meeting with the lead researcher to review the results. Interviews were conducted by the first author (EH).

### Data analysis

Reflexive thematic analysis (RTA) is a theoretically flexible approach to qualitative data analysis (Braun & Clarke, 2019). An inductive approach within a critical realist framework was used, whereby coding and theme development were directed by the data content, with an assumed reality within the data requiring interpretation to access the underlying structures (Braun & Clarke, 2021; Willig, 2012). Note that the questionnaire measure scores were not incorporated into the analysis.

Braun and Clarke's (2021) six‐phase guide of RTA was used to analyse the data, comprising of: (1) transcribing, reading and re‐reading interviews to generate initial ideas; (2) systematic data coding; (3) generating potential themes from codes; (4) developing and reviewing themes; (5) refining and naming themes; and (6) writing the analysis. The analysis was a recursive process involving movement back and forth between phases. Interviews were transcribed by the lead researcher, and accuracy alongside the audio recordings was checked throughout. The lead researcher undertook the analysis, and this was finalised through discussions with the research team to ensure the trustworthiness of the findings (Krefting, 1991).

The lead researcher (trainee clinical psychologist) was a white British woman with no personal NSSI experience but a strong professional interest in this area. They had previously completed an undergraduate degree in psychology and worked in both clinical and research posts within mental health. The second and third authors were clinical psychologists and researchers with clinical expertise working with individuals who have self‐injured and academic experience of qualitative research.

## RESULTS

### Participant characteristics

Eighteen people agreed to participate in the study. One person was excluded due to not meeting the inclusion criteria. One participant withdrew from the study at the start of the interview due to difficulty discussing self‐injury. The risk protocol was followed, and the participant was signposted to further support (a copy of the risk protocol is available upon reasonable request). This resulted in a total of 16 participants.

Participant characteristics are summarised in Table 1. The ages of participants ranged from 21 to 61 years (*M =* 30.5), and half of the participants described themselves as white British (*n =* 8). Information regarding NSSI was obtained from the SITBI to help characterise the sample. The age of NSSI onset ranged from 7 to 21 years (*M =* 14.1). All participants were highly educated, with the majority completing a minimum of an undergraduate degree. NSSI occurred over an average of 13.4 years (range 2–51 years). Participants struggled to give an estimate for lifetime NSSI, with estimates ranging from 20 to 1000 (with one person saying they had “lost count”). Participants reported a range of self‐injury methods, predominantly cutting or burning skin. Most participants (*n =* 13) had previously accessed services regarding their self‐injury. Services regarded as ‘other’ included occupational therapy, GP, and charities. Three participants had not accessed any services relating to their self‐injury. Thirteen participants described themselves as no longer self‐injuring at the time of the interview. Two participants had self‐injured within the past month, and one participant self‐injured within the past week; however, these participants perceived a notable improvement, hence meeting the inclusion criteria. The mean QPR score for participants was 44.9 (SD = 7.7), similar to previous definitions of personal recovery as a QPR score of 45 or higher (Best et al., 2020). Higher QPR scores indicate greater perceived recovery.

**TABLE 1:** Participant characteristics.

Characteristics	*n* (%)
Age	
20–29	10 (63)
30–39	4 (25)
40–49	1 (6)
60–69	1 (6)
Ethnicity	
White British	8 (50)
White Asian	1 (6)
White European	2 (13)
Black African	1 (6)
Black British	1 (6)
British Iranian	1 (6)
White Other	2 (13)
Gender	
Female	15 (94)
Transgender male	1 (6)
Sexual orientation	
Heterosexual	8 (50)
Gay or lesbian	2 (13)
Bisexual	4 (25)
Other	1 (6)
Prefer not to answer	1 (6)
Relationship status	
Single	9 (56)
Married	2 (13)
In a relationship	5 (31)
Education level	
College/Sixth Form	2 (13)
Vocational training	1 (6)
Undergraduate degree	7 (44)
Master's degree	3 (19)
Doctorate/PhD	3 (19)
Employment status	
Student	2 (13)
Part‐time employed	2 (13)
Full‐time employed	8 (50)
Unemployed	4 (25)
Services accessed in relation to NSSI	
Psychological therapy	11 (69)
Medication	9 (56)
Community mental health team	9 (56)
Crisis intervention	7 (44)
Inpatient hospital treatment	6 (38)
Medical treatment	4 (25)
Other	9 (56)

### Member‐checking

Fourteen participants who provided consent were invited to separate member‐checking meetings with the lead researcher. Two participants attended and were given the opportunity to comment on and validate the preliminary findings (Doyle, 2007). Participants responded positively to the member‐checking meeting, commenting that participating in the study and seeing the culmination of theirs and others' contributions was a rewarding, moving and informative process. Participants confirmed the credibility of the results.

### Themes

Four core themes were identified from the analysis: ‘increased understanding of self‐injury experiences,’ ‘social connection and belonging,’ ‘finding the right strategies,’ and ‘moving towards a life I want’.

### Theme one: Increased understanding of self‐injury experiences

Developing personal understanding of their own self‐injury, life experiences, and psychosocial stressors improved difficulties with self‐injury in several ways. It allowed individuals to view themselves and their self‐injury more compassionately and encouraged self‐acceptance. Increased insight supported reflection on the negative unintended consequences of self‐injury, motivating holistic change.

#### Compassionate insight and self‐acceptance

Participants described a process of making sense of their self‐injury within the context of their life experiences through gradual attainment of psychological distance and self‐reflection: “I think it's just getting older and looking back at all the things that were going on; it kind of, it just makes sense, you know” (P17). Connecting to others with shared experiences helped participants develop insight and self‐awareness of personal triggers and recognise emotional patterns. Through making sense of their experiences, participants acknowledged self‐injury as a valid response to their circumstances, allowing a more compassionate view of themselves and reducing blame: “I've learnt more about myself and stuff that happened to me, and the main thing is that I've realised none of it was my faul” (P13). Acknowledging and validating past difficulties helped participants move towards self‐acceptance, “I don't like [self‐injury], but I don't fight against it as sort of being part of me anymore” (P4), becoming more accepting that self‐injury does not define them, and they can live alongside NSSI; “It's like I used to share a room with self‐injury, but now it lives next door” (P5). Compassionate insight and self‐acceptance enhanced self‐worth, encouraging some participants to reduce self‐injury: “I don't want to punish myself like that anymore” (P3). For others, they viewed themselves more compassionately at times of distress when they may still self‐injure, reducing feelings of guilt: “I don't beat myself up when it happens, which sounds ironic when you're talking about self‐injury. I don't chastise myself mentally or emotionally about it anymore” (P4). Participants that accessed services for self‐injury support often received little compassion; “…feeling shit enough to self‐injure and then going to get some medical attention and them treating you like shit is not really that really helpful” (P13).

#### An understandable response with unintended consequences

Comprehending self‐injury as an understandable response to adverse experiences helped participants validate self‐injury as a coping strategy when little else was available and highlighted how self‐injury had helped them through difficulty: “[self‐injury] got me through quite a lot, and I kind of needed that at the time” (P17). Increased insight into self‐injury enabled participants to reflect on its dualism, balancing the perceived positive and negative aspects of self‐injury and beginning to recognise the broad impact on oneself and others: “[Self‐injury] was helpful to me because it was like an expression of the intense emotions I was feeling. But it got to the point where it crossed over to being more harmful because of the long‐term healing process that I'd have to go through” (P3). Participants recognised various negative consequences of self‐injury. It was noted that physical scars might impede life goals. Recognising the emotional impact on close relationships motivated participants to initiate change, “I know if I do it my boyfriend immediately will know what it is and what it looks like, so I don't want to sort of upset the people that I care about” (P5), as did recognising potentially irreversible health consequences and risk of unintended death; “I mean I did nearly die. Which I think was like a kind of a bit of a wake‐up call. But people had been telling me for ages that, like, it doesn't matter whether that's what you intend or not; like, sooner or later, that's what's going to happen” (P6). Considering the opposing impacts of self‐injury highlighted that the negative unintended repercussions outweighed the positive, instigating change.

### Theme two: Social connection and belonging

Establishing support networks with fulfilling reciprocal relationships was fundamental to improving self‐injury, enhancing inclusion, belonging, and sense of safety. Developing positive relationships with like‐minded people helped some participants feel valued and connected to others: “When I went to university, there were sort of people who were just much more at a level that I could relate to, and I found it much easier to build relationships that were positive and things; there wasn't any one particular one” (P2). This helped alleviate loneliness and improved self‐esteem: “…I started developing a lot better relationships with people; I stopped feeling so lonely and anxious and just felt better about myself, I think” (P15). Unconditional support helped participants feel safe to talk about difficulties without fear of judgement: “…having a person you can talk to about what's going on in your life in a way that's not, like, they care equally about you whether you're doing well or whether you're doing badly; it makes a massive difference” (P6). Acceptance from others helped participants move towards self‐acceptance, feeling safe to be their authentic selves and improving their relationship with self‐injury. Contrastingly, participants that accessed services often felt unheard: “I didn't really feel listened to; it kind of, I just felt like I learnt more about the lady sat opposite me than I felt like I got out of it” (P17). Lack of continuity within services made it difficult to build trusting relationships; “It's very stressful to try and explain everything over and over again, especially when you get to my age” (P3).

### Theme three: Finding the right strategies

Participants reported gaining alternative coping strategies and enhanced control over their self‐injury. When faced with situations where previously they may have self‐injured, they had more available options without negative unintended consequences. Distraction techniques such as focusing on work, taking a walk, or creative activities helped some participants shift their attention from negative thoughts, allowing difficult feelings to subside and reducing the desire to self‐injure: “But if I can do something else, like work, I start switching more into like logic and reason, and I'm focusing on something else. So that gives me a distraction; it gives me a puzzle to solve, which just takes my mind off, it and then the moment has passed, and I can calm down a bit then” (P5). Using alternative methods to express difficult emotions was helpful for some participants, such as journaling. Other participants found acting out on their emotions, but not causing damage to themselves, was helpful to release initial intense emotions, allowing subsequent reflection and helpful action: “…I will just throw something. I'll just vent the anger, and then I'll go and sit down for a minute, and I'll just sort of think about what's going on, and like, is this how I want to react to it?” (P3). Through developing insight into their self‐injury, some participants separated thoughts about self‐injury from acting on thoughts: “I've had times where maybe I had like a bad breakup, where my brain did instantly go to [self‐injury], but I'm much better at not acting on that now” (P14). This helped participants feel more in control and allowing self‐injurious thoughts to pass: “…I guess when I feel like that now, it's easier to ride it out because I kind of know that it's not permanent” (P15).

These strategies were personal and often a process of trial and error. Many participants felt comforted that self‐injury remained in the background if they ‘needed’ it. However, some participants were clear self‐injury was no longer an option, emphasising the personal nature of ‘recovery’. Despite developing alternative coping strategies, some participants expressed concern that services would remove their ‘safety net’ by stopping their self‐injury: “…at the time, [self‐injury] wasn't something that I wanted to stop doing, because it was the thing that was helping… so I wouldn't have wanted anybody to say, ‘Oh, let's stop doing this’…” (P2).

### Theme four: Moving towards a life I want

Many participants described rebuilding a life they wanted for themselves. Participants began to acknowledge and prioritise their needs and make choices consistent with their hopes and values; “…I suppose what I did was get rid of all pressures and all pulls on my time and just work out what was helpful to us” (P8). For some, involvement with meaningful and valued roles (e.g., volunteering) improved self‐esteem. For others, using their lived experience to help others, raise awareness, or influence systems created purpose and motivation: “I'm studying to be a mental health nurse because I want to use my experiences to help other people” (P3). Several participants valued reconnecting with their interests and participating in recreational activities which enriched their lives; “I got to a place where I had other things that were more important and other things that I could enjoy and, like, not pour myself into but, like, invest in and enjoy and find purpose in, and when things were hard, like, have that rather than self‐harm” (P1).

Many participants reported the significance of changing their physical and social environment to better suit their needs in improving difficulties with self‐injury: “I'm more settled in [location] like; having moved away from home was like a big change, whereas now I'm happy where I am” (P5). Moving from detrimental environments where individuals felt controlled or isolated to more stable environments where they had more freedom to make life choices increased autonomy and independence and enhanced overall well‐being; “Once I got out into university, you just‐you're able to choose when you're with people or even spend time on your own, and you know who you want to spend the time with. You can just leave if you don't like it” (P2).

## DISCUSSION

The current study aimed to investigate personal experiences of naturalistic improvements in NSSI, given the limited research on this phenomenon. A reflexive thematic analysis of participant interviews revealed four main themes. Increased insight into self‐injury experiences validated self‐injury and promoted self‐compassion and self‐acceptance while acknowledging the negative unintended consequences of NSSI. Safe and supportive relationships alleviated loneliness, and developing alternative coping strategies enhanced control over self‐injury. Living within one's values promoted independence, choice, and self‐esteem.

The results demonstrate that people who self‐injure can and do experience naturalistic improvement with NSSI, influenced by a variety of internal and external factors, similar to the naturalistic recovery processes outlined by Onken et al. (2002) for mental health difficulties more generally. In the current study, developing a deeper understanding of their self‐injury and past experiences helped curate self‐compassion and self‐acceptance, reducing blame and promoting care for oneself. This supports findings by Sutherland et al. (2014) whereby cultivating greater self‐compassion and acceptance of one's NSSI experiences ameliorated distress and fostered improvements with NSSI. Other research has demonstrated an inverse association between self‐compassion and NSSI, supporting this link (Per et al., 2022; Suh & Jeong, 2021). Similarly, Shaw (2006) found developing psychological insight and valuing oneself were essential towards ceasing self‐injury. However, it was not considered how compassion could be implemented alongside continued engagement with self‐injury. Research indicates self‐compassion can promote psychological well‐being, protect against distress, and increase life satisfaction (Neff, 2003; Neff et al., 2007), and compassion and acceptance are treatment goals for some therapies (e.g., compassion‐focused therapy; Van Vliet & Kalnins, 2011). Therefore, compassion and acceptance‐based frameworks may prove useful for those who self‐injure, and levels of self‐compassion may be a more meaningful indicator of naturalistic improvements in NSSI than cessation of the behaviour.

Prior research found that recognising the negative consequences of self‐injury (e.g., scarring, impact on loved ones) compelled self‐injury cessation (Buser et al., 2014; Gelinas & Wright, 2013; Shaw, 2006). The current study findings highlighted participants' awareness of the conflict between viewing NSSI as helpful and unhelpful. Over time, recognising that negative unintended repercussions outweighed perceived benefits motivated holistic change through seeking alternative coping strategies or becoming involved in meaningful roles, as opposed to solely motivating behavioural cessation. This could be seen as a shift from a pre‐contemplative to a contemplative stage of change (and subsequently on to action; Kruzan & Whitlock, 2019). The benefit and barriers model (Hooley & Franklin, 2018) of self‐injury suggests NSSI may have a variety of relatively universal benefits (e.g., emotion regulation), which are balanced by a series of barriers (e.g., fear around harming oneself). In those who self‐injure, it is suggested that these barriers have been weakened. Re‐establishing ‘barriers’ such as a more positive perception of the self may be an important treatment target. Hooley and Franklin (2018) suggest that a positive sense of self is an important barrier to NSSI (see also Forrester et al., 2017), and hence therapeutic work to improve this, such as cognitive‐behavioural work on self‐esteem and core beliefs, may contribute to recovery.

Several participants attributed improvements with their self‐injury to establishing trusting and supportive social connections, whereby they felt accepted, valued, and listened to. This is supported by previous findings that improved social relationships and open, non‐judgemental interactions facilitated NSSI ‘recovery’ (Buser et al., 2014; Meheli et al., 2021; Shaw, 2006). Developing such skills to improve relationship quality between individuals who self‐injure and their families or peers may be a key area for intervention (Peel‐Wainwright et al., 2021; Tatnell et al., 2014). Proactive and accessible resources for families and peers of those who self‐injure may be useful to facilitate self‐injury awareness and encourage the implementation of valuable and practical support strategies (e.g., active listening, non‐judgemental approach; Curtis et al., 2018). Additionally, lack of perceived belonging has been linked to NSSI (Dunlop et al., 2022), and the current study found meeting socially similar individuals, or individuals sharing similar experiences, enhanced belongingness, reduced isolation, and subsequently improved their relationship with NSSI (Boyce et al., 2018; Thoits, 1986).

Although some participants considered completely stopping self‐injury as inconceivable, developing a sense of control over self‐injurious behaviours was important. Acquiring alternative coping mechanisms helped participants feel less overwhelmed by self‐injury and better able to make choices about NSSI engagement due to having more available options during distress. The current study highlighted the individual nature of effective strategies. Participants described developing greater psychological flexibility, whereby compassionate understanding and non‐judgemental awareness of internal and external experiences enabled participants to monitor their emotions and guide their cognitions and behaviours in more helpful ways, rather than progressing to NSSI (Neff, 2003), and acknowledging difficult thoughts will subside. Greater psychological flexibility has been associated with a range of positive psychological, interpersonal, and health‐related outcomes, including more effective coping with psychological trauma and distress (Kashdan & Rottenberg, 2010), although issues with the way psychological flexibility has often been measured have been highlighted (Doorley et al., 2020). Psychological flexibility may therefore be an important intervention target.

Participants in the current study described that rebuilding a life consistent with their hopes and values was instrumental in alleviating difficulties with self‐injury. Reconnecting with interests and participating in meaningful roles provided a sense of achievement, purpose, and improved self‐esteem (Shaw, 2006; Wills, 2012). Many participants described moving away from the stressors within their home environment to environments where they gained independence and autonomy, which allowed them more freedom to make autonomous decisions and enforce personal boundaries. Similarly, Kool et al. (2009) found that increased autonomy gave individuals more control over forming personal connections. While many participants discussed rebuilding a ‘good enough’ life for their individual needs, it is important to consider that many individuals continue to experience intolerable social and economic conditions (e.g., poverty, stigma, racism, sexism; Recovery in the Bin, 2023). Therefore, rebuilding a life in line with one's hopes and values may be unachievable for many without social, political, and economic change.

There were limitations to the current study. Although the sample of participants captured a range of ethnicities, ages, and LGBTIQ+ individuals, it comprised mostly highly educated females. Given NSSI also affected many men (Heath et al., 2008; Hilt et al., 2008; Staring et al., 2024), elevated NSSI prevalence among gender minorities (Liu et al., 2019), and the association between unemployment and deprivation with self‐harm (Ayton et al., 2003), views from these groups should be explored in future research. Additionally, there are limitations to conducting interviews by video call, including potential difficulties judging non‐verbal behaviour, possible initial discomfort adapting to video calls, and increased focus on one's own behaviour may impede communication (Payne et al., 2020). Nevertheless, evidence suggests video calls can increase accessibility (Thomas et al., 2021) and promote openness and disclosure (Fernández‐Álvarez & Fernández‐Álvarez, 2021). There is debate around the value of member checking that should be acknowledged. Whilst this may provide further insights into themes or allow additional contextualisation of findings, it can also lead to challenges where participants disagree with their earlier accounts and wish these to be modified (Goldblatt et al., 2011). The power imbalance between researcher and participant may also have affected the utility of member checking by limiting what participants felt able to disagree with or challenge (Goldblatt et al., 2011). In the present study, only two participants attended the member checking, limiting its value as a check of the trustworthiness or validity of findings.

An inclusion criterion for the study was that individuals who had accessed therapy or medication did not attribute improvements in their NSSI to these treatments. As this was based on self‐report, a possibility remains that some of these participants may still have gained from these treatments but have not realised this or misattributed the reason for their improvements. In such cases it is arguably still important to understand the participant's perception of what helped, as they provide a useful insight into the participants' understanding of their recovery, but nonetheless this issue should be held in mind. Whilst we refer to the analysis as inductive, it should be noted that the analysis was constructed through the lens of which the data is viewed, informed by the literature and the researcher's own experiences, assumptions, and knowledge (Braun & Clarke, 2019).

Consistent with person‐centred models of self‐injury recovery (Lewis & Hasking, 2020), participants described a range of holistic internal and external factors contributing to naturalistic improvements in NSSI. Despite this, many participants' experiences of health services regarding self‐injury directly contrasted these. Although NICE guidelines (2022) state people accessing services with self‐harm should be treated with compassion and respect, many participants described experiencing judgemental and uncompassionate responses from health professionals impeding opportunities to build safe and trusting relationships with professionals, and exacerbating isolation (Rosenrot & Lewis, 2020; Sykes et al., 2015). Consideration should be given to how health services can embed the approaches identified into their care; for example, approaches rooted in self‐compassion may improve outcomes for individuals that self‐injure (Sutherland et al., 2014). Interventions that emphasise behavioural cessation may overlook the underlying issues and psychosocial context within which self‐injury develops (Long et al., 2016) and ignore the multifaceted aspects deemed meaningful to individuals, and therefore be limited in effect. The current study findings highlight the importance of actively listening to and valuing individual wants and needs to develop meaningful person‐centred goals, which may reduce unrealistic expectations from prioritising behavioural cessation (Lewis & Hasking, 2020). Future research is needed to further understand how these naturalistic processes can be integrated into NSSI treatments and support.

Whilst this study focused on experiences that were not attributed to therapy or medication, there are notable parallels with what people who self‐harm find beneficial within psychological therapy (see Haw et al., 2022). In particular, acceptance, both of self and one's self‐harm, appears central, alongside having trusting relationships (therapeutic or otherwise) within which one feels safe to share and is able to be validated. Such parallels are perhaps not surprising and may speak to the importance of ‘common factors’ key to recovery that can occur in psychotherapy or through naturalistic means (Wampold, 2015). Clinically, attending to non‐specific factors and creating a safe and trusting therapeutic relationship where clients feel heard and understood, therefore, is important. More broadly, given the relevance of relationships in participants' lives, relational therapeutic approaches that attend to these wider relationships and how participants navigate them (e.g., cognitive analytic therapy; Taylor et al., 2024) may be helpful. Likewise, helping clients to work towards valued or personally meaningful goals that help them ‘move towards the life they want’. These suggestions are consistent with recommendations that psychotherapy for NSSI needs to see the person beyond the behaviour (Haw et al., 2022).

The current study contributes to the literature by highlighting personal and multifaceted factors and experiences deemed helpful in improving difficulties with NSSI, extending beyond a focus on NSSI cessation. This has important implications for considering how health services can embed these factors into their support to improve outcomes for individuals that self‐injure. Due to the personal nature of the factors supporting naturalistic improvements with NSSI, it is not expected that a single alternative is suitable for everyone.

## AUTHOR CONTRIBUTIONS

**E. Hudson:** Conceptualization; investigation; methodology; data curation; formal analysis; writing – review and editing; writing – original draft; project administration. **S. Hartley:** Conceptualization; writing – review and editing; supervision. **P.J. Taylor:** Writing – review and editing; supervision; conceptualization; formal analysis.

## FUNDING INFORMATION

No funding was provided for this research.

## CONFLICT OF INTEREST STATEMENT

None.

## Supporting information


Table S1:

## Data Availability

Research data are not shared.
