# Supplementary material for: “It's like I used to share a room with self‐injury, but now it lives next door”: Exploring experiences of naturalistic improvement in non‐suicidal self‐injury
Source: Psychol Psychother. 2024 Dec 19;98(1):133–48. doi: 10.1111/papt.12567 (PMC11823337; doi:10.1111/papt.12567)
Supplement: Supplementary file 1 — Table S1: [file PAPT-98-133-s001.docx]

**Supplementary Table 1**

**Consolidate criteria for Reporting Qualitative Research (COREQ; Tong et al., 2007) checklist**

| Domain | Details |
| --- | --- |
| Domain 1: Research team and reflexivity |  |
| 1. Interviewer/facilitator Which author/s conducted the interview or focus group? | All interviews undertaken by first author (see Procedure; Page 7) |
| 2. Researcher credentials | see Procedure (Page 7) |
| 3. Researcher occupation | see Procedure (Page 7) |
| 4. Researcher gender | see Procedure (Page 7) |
| 5. Researcher experience and training | As a trainee clinical psychologist, the lead researcher was receiving doctoral level training in research methods and psychological understandings of mental health. |
| 6. Relationship established with participants | see Procedure (Page 7) |
| 7. Participant knowledge of the interviewer | Participants had no personal knowledge of the researcher prior to the study, but the rationale and the focus of the research was shared in the participant information sheet before consent was sought and all participants had the chance to ask questions. |
| 8. Interviewer characteristics | See Data Analysis (Page 7). The research team held a predominantly social and psychological understanding of the causes of self-injury. The focus of the research was driven by an awareness, developed through speaking with people who had self-inured that many had experienced improvements in their difficulties that were not attributed to formal psychotherapy or medication. The researchers were aware of a lack of research in this area and interested in exploring this topic. |
| Domain 2: study design |  |
| 9. Methodological orientation and theory | See Data Analysis (Page 7) |
| 10. Sampling | See Participants (Page 5) and Procedure (Page 7). |
| 11. Method of approach | See Participants (Page 5) and Procedure (Page 7). |
| 12. Sample size | See Participants (Page 5) |
| 13. Non-participation | See Participants (Page 5) |
| Setting |  |
| 14. Setting of data collection | See Procedure (Page 7). |
| 15. Presence of non-participants | No other individuals were present during interviews. |
| 16. Description of sample | See Participants (Page 5) |
| Data collection |  |
| 17. Interview guide | See Interviews (Page 6) |
| 18. Repeat interviews | Only a single interview was undertaken, but member checking was employed as noted (see Results; Page 8). |
| 19. Audio/visual recording | See Interviews (Page 6). |
| 20. Field notes | A reflective log was kept by the lead author, interview and analyst (EB). |
| 21. Duration | See Interviews (Page 6). |
| 22. Data saturation Was data saturation discussed? | The concept of data saturation has been challenged in recent years and was not employed within this study as a criterion for determining sample size (e.g. Malterud et al., 2016)  Malterud, K., Siersma, V. D., Guassora, A.D. (2016). Sample size in qualitative interview studies: Guided by information power. Qualitative Health Research. 26, 1753-1760. doi:10.1177/1049732315617444 |
| 23. Transcripts returned | Transcripts were not shared with participants. |
| Domain 3: analysis and findings |  |
| 24. Number of data coders | The first author undertook all the coding |
| 25. Description of the coding tree | The analysis process including coding is described (see Data Analysis; Page 7) but full details of codes generated are not shared due to space limitations. |
| 26. Derivation of themes | Themes were derived from the data (see Data Analysis; Page 7) |
| 27. Software | No software was used. |
| 28. Participant checking | See Member-checking (Page 9) |
| 29. Quotations presented | See Results (Page 9) |
| 30. Data and findings consistent | Yes, See Results (Page 9) |
| 31. Clarity of major themes | See Results (Page 9) |
| 32. Clarity of minor themes | See Results (Page 9) |
